# Prevalence of mutations in inherited retinal diseases: A comparison between the United States and India

**DOI:** 10.1002/mgg3.1081

**Published:** 2019-12-09

**Authors:** Sophia Yohe, Malaichamy Sivasankar, Anuprita Ghosh, Arkasubhra Ghosh, Jennifer Holle, Sakthivel Murugan, Ravi Gupta, Lisa A. Schimmenti, Ramprasad Vedam, Bharat Thyagarajan

**Affiliations:** ^1^ Department of Laboratory Medicine and Pathology University of Minnesota Minneapolis MN; ^2^ Medgenome Labs Ltd. Bangalore India; ^3^ Narayana Nethralaya Bangalore India; ^4^ Present address: Invitae San Francisco CA; ^5^ Present address: Mayo Clinic Rochester MN

**Keywords:** clinical testing, molecular diagnosis, next‐generation sequencing, racial disparity, retinal disorders

## Abstract

**Background:**

Studies evaluating next‐generation sequencing (NGS) for retinal disorders may not reflect clinical practice. We report results of retrospective analysis of patients referred for clinical testing at two institutions (US and India).

**Methods:**

This retrospective study of 131 patients who underwent clinically validated targeted NGS or exome sequencing for a wide variety of clinical phenotypes categorized results into a definitive, indeterminate, or negative molecular diagnosis.

**Results:**

A definitive molecular diagnosis (52%) was more common in the India cohort (62% vs. 39%, *p* = .009), while an indeterminate molecular diagnosis occurred only in the US cohort (12%). In the US cohort, a lower diagnostic rate in Hispanic, non‐Caucasians (23%) was seen compared to Caucasians (57%). The India cohort had a high rate of homozygous variants (61%) and different frequency of genes involved compared to the US cohort.

**Conclusion:**

Despite inherent limitations in clinical testing, the diagnostic rate across the two cohorts (52%) was similar to the 50%–65% diagnostic rate in the literature. However, the diagnostic rate was lower in the US cohort and appears partly explained by racial background. The high rate of consanguinity in the Indian population is reflected in the high rate of homozygosity for pathogenic mutations and may have implications for population level screening and genetic counseling. Clinical laboratories may note diagnostic rates that differ from the literature, due to factors such as heterogeneity in racial background or consanguinity rates in the populations being tested. This information may be useful for post‐test counseling.

## Graphical abstract

Hispanic/non‐White patients had a lower diagnostic rate for retinal disorders on NGS testing and the Indian cohort had a higher rate of homozygous variants. Heterogeneity in racial background may lead to different diagnostic rates within individual populations, which may be important for patient counseling.

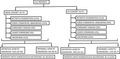

## 1 INTRODUCTION

Inherited retinal diseases are a heterogeneous group of syndromic and non‐syndromic diseases with variable inheritance caused by mutations in over 200 genes that lead to overlapping phenotypes (Daiger, Sullivan, & Bowne, 1998). Hence, testing of large panels of genes by next‐generation sequencing (NGS) is a cost effective method to evaluate patients with suspected inherited retinal disease (Audo et al., 2012; Bernardis et al., 2016; Glockle et al., 2014; Riera et al., 2017).

Studies have shown that NGS of gene panels leads to higher detection of a genetic cause for retinal disease than traditional methods such as Sanger sequencing; however, these studies often have features that do not reflect clinical practice and may prevent extrapolation of reported diagnostic rates to clinical practice. Some studies recruited patients and often their families, while others heavily weighed in silico predictions for classifying missense variants as pathogenic and only one of these studies took into account American College of Medical Genetics and Genomics (ACMG) criteria for classifying variants, which puts low value on in silico predictions (Audo et al., 2012; Bernardis et al., 2016; Carss et al., 2017; de Castro‐Miro et al., 2016; Glockle et al., 2014; O'Sullivan et al., 2012; Riera et al., 2017). Some studies investigated only subsets of patients or patients with well‐defined phenotypes (Audo et al., 2012; Licastro et al., 2012; O'Sullivan et al., 2012). In clinical practice, family members may not be available for segregation studies, patients may have had prior testing, phenotypes may not be classic or well defined, and functional studies are not performed to help variant classification. Finally, most of the studies included only Caucasian participants with homogenous racial background and the results may not be representative for racially diverse populations commonly seen in clinical practice. Finally, several previous reports have identified lower mutational frequency of specific genes in South Indian populations as compared to Western populations, which suggested that diagnostic yield of commonly used targeted NGS panels may be lower in the Indian population as compared to Western populations (Joseph et al., 2002; Lotery et al., 2000; Sundaresan et al., 2009). For these reasons, we undertook a retrospective study at two institutions in the US and India with racially heterogeneous patient populations who underwent clinical NGS testing for inherited retinal disorders with all its inherent limitations to determine the clinical utility of genetic testing in retinal disorders.

## 2 MATERIALS AND METHODS

In this retrospective study approved by the institutional review boards at the University of Minnesota (US cohort) and Narayana Nethralaya, Bangalore, India (Indian cohort), we identified 131 probands (57 US and 74 Indian) with retinal dystrophy who underwent clinical genetic testing using NGS between January 2013 and March 2017. OMIM numbers and reference transcripts using GRCh37 are given in the Table S1.

**Figure 1:**
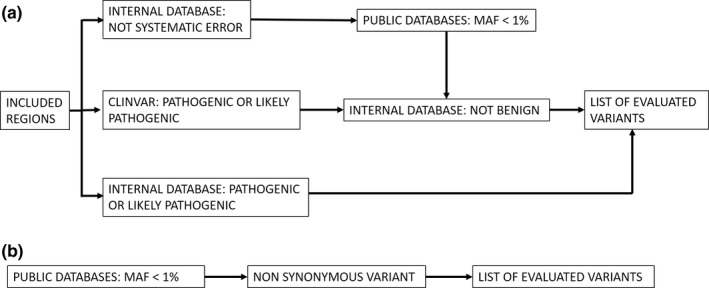
Filtering strategies for raw variant call file (vcf) to list of evaluated variants for the (a) US and (b) Indian cohorts. (a) In the US cohort, the vcf was filtered for included regions. Any variant previously categorized as pathogenic or likely pathogenic was added to the list of evaluated variants. Variants with a minor allele frequency (<0.01) that were not systematic error and not previously categorized as benign internally were also added to the list of evaluated variants. Finally, to avoid missing common pathogenic variants, any pathogenic or likely pathogenic variant in ClinVar (including conflicting interpretations) that were not previously categorized as benign internally were added to the list of evaluated variants. (b) In the Indian cohort, any nonsynonymous variant with an maf equal to or less than 0.01 by 1,000 Genomes, Exac, EVS, DBSNP147, 1,000 Japanese Genomes, or an internal Indian database were evaluated

All patients from the US were tested using a targeted NGS panel ranging from 10 genes to 257 genes based on clinical presentation. In most cases (51 of 57 cases, 89%), 175 or more genes were tested. From August 2012 to March 2014, target enrichment was performed using a custom SureSelect (Agilent Technologies Inc.) panel of 568 genes (1.84 MB genomic region), followed by sequencing on a HiSeq2000 (Illumina Inc.) and clinically ordered genes were analyzed using a custom bioinformatics pipeline as previously published (Yohe et al., 2015). For cases after March 2014, target enrichment was performed using TruSight One (Illumina Inc.) panel of 4,871 genes (10.5 MB genomic region), followed by sequencing on a HiSeq2500 rapid‐run mode (Illumina Inc.) using a custom bioinformatics pipeline (Nelson et al., 2015). A minimum coverage of 20× was targeted with an average coverage around 200×. The variant call file (vcf) was filtered using minor allele frequency (maf), prior categorization from our internal database, and categorization in ClinVar. (Figure 1a).

The Indian patients were tested at MedGenome for a targeted NGS panel (62 cases) or by whole exome sequencing (12 cases). Initially, the targeted panel consisted of a custom capture (Roche NimbleGen) of 200 genes (0.8 Mb genomic region) associated with retinal phenotypes. In 2015, this was updated to a clinical exome of around 7,000 genes (26 MB) and retinal genes (based on RETNET) were analyzed (Daiger et al., 1998). The libraries were sequenced for a mean coverage >80–100× on an Illumina sequencer. Whole exome sequencing was performed using Agilent Sureselect V5 (Agilent Technologies Inc) and sequenced on an Illumina sequencer. Sequences were aligned to human reference genome (GRCh37/hg19) using BWA [1, 2] and analyzed using Picard, GATK lite, and GATK version 3.6(Broad Institute, Cambridge, MA) [3, 4] using GATK best practices framework for identification of variants in the sample. Gene annotation of the MODY and neonatal diabetes gene variants is performed using VEP program [5] against the Ensembl release 87 human gene model [6]. Clinically relevant mutations were annotated using published variants in literature and a set of diseases databases—ClinVar, OMIM, GWAS, HGMD, and SwissVar [7–14]. Nonsynonymous variant effect is calculated using multiple algorithms such as PolyPhen‐2, SIFT, MutationTaster2, Mutation Assessor, and LRT. The vcf was filtered using GeneInsight (Sunquest Tuscon) based on maf and non‐synonymous changes. (Figure 1b).

Clinical information, including age, gender, retinal phenotype, family history, and consanguinity, was obtained from the medical records. ACMG criteria were used by both institutions to classify variants as pathogenic (P), likely pathogenic (LP), or variants of uncertain significance (VUS) (Richards et al., 2015). Although several variants were classified as VUS using the ACMG criteria, only a subset of VUS (suspicious VUS) are reported here. These include (a) VUS present in a gene with a P or LP variant, (b) VUS with a higher likelihood of being pathogenic based on the clinical context and variant type, and (c) variants with controversial significance in the medical literature. For the Indian cohort, suspicious VUS were not reported if there was other evidence of a molecular diagnosis.

A definitive molecular diagnosis was defined as a single P or LP variant for an autosomal dominant disorder or X‐linked disorder in a male. For autosomal recessive disorders, two P, two LP, or a single P and a single LP variant in the same gene were required for a definitive molecular diagnosis. Cases of autosomal recessive disorders with one P or LP variant and one VUS in the same gene were defined as an indeterminate molecular diagnosis (Glockle et al., 2014). Other combinations were considered negative for a molecular diagnosis. Values of *p* were calculated using a two‐tailed Fisher's exact test using the statistical program QuickCalcs (https://www.graphpad.com/quickcalcs/).

## 3 RESULTS

The average age of patients in both cohorts was 17.9 ± 16.4 years with a range of less than 1 year to 70 years with similar sex ratios in both cohorts. (Table 1) The US cohort was predominantly non‐Hispanic White, (44 cases, 77%) while 13 cases (23%) had at least 50% Hispanic and/or non‐White ancestry. The ethnicities in this group were diverse: Mexican (3), Somali (3), Native American (2), Korean (1), Pakistani (1), Hmong (1), Burmese (1), and Puerto Rican (1). Of the 54 cases from India where ancestry was known, 43 (80%) were from South India, 8 (15%) were from North India, and 3 (5%) were from Central India.

**Table 1:** Distribution of clinical and molecular diagnoses in the Indian and US cohorts

Characteristics	India	US	Total	*p* value
Total number of cases	74 (56%)	57 (44%)	131	
Male	46 (62%)	29 (51%)	75	.22
Female	28 (38%)	28 (49%)	56	
Age (average ± *SD*)	17.6 ± 14.7	18.3 ± 18.5	17.9 ± 16.4	.74
Homozygous variants	45 (61%)	7 (12%)	52 (0%)	<.0001
Definitive molecular diagnosis (number (%))	46 (62%)	22 (39%)	68 (52%)	.009
Possible molecular diagnosis (number (%))	0 (0%)	7 (12%)	7 (5%)	
Molecular diagnosis not made (number (%))	28 (38%)	28 (49%)	56 (43%)	
Clinical phenotype				
Retinitis pigmentosa	22 (30%)	17 (30%)	39 (30%)	
Definitive molecular diagnosis	14 (64%)	5 (29%)	19 (49%)	
Possible molecular diagnosis	0	4 (24%)	4 (10%)	
Leber congenital amaurosis	17 (23%)	5 (9%)	22 (17%)	
Definitive molecular diagnosis	14 (82%)	4 (80%)	18 (82%)	
Possible molecular diagnosis	0	0	0	
Stargardt disease	7 (9%)	0	7 (5%)	
Definitive molecular diagnosis	4 (57%)	0	4 (57%)	
Possible molecular diagnosis	0	0	0	
Usher syndrome	0	4 (7%)	4 (3%)	
Definitive molecular diagnosis	0	1 (25%)	1 (25%)	
Possible molecular diagnosis	0	0	0	
Other clinical diagnosis	28 (38%)	31 (56%)	59 (46%)	
Definitive molecular diagnosis	14 (52%)	12 (38%)	26 (43%)	
Possible molecular diagnosis	0	3 (9%)	3 (5%)	

**Figure 2:**
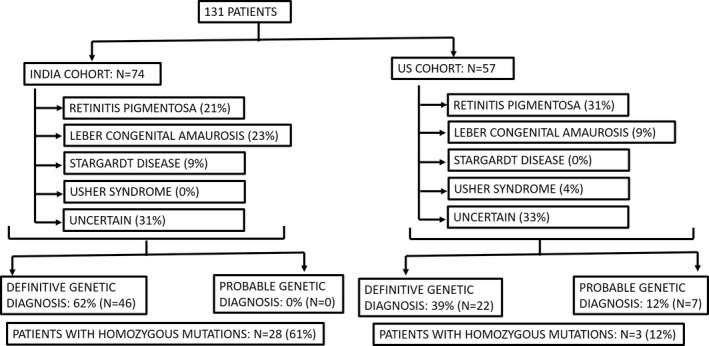
There were 131 patients across both cohorts (57 US, 74 Indian). Percentages of each cohort with select clinical diagnoses are listed for comparison, as are the number of definitive and probable genetic diagnoses and homozygous mutations

Retinitis pigmentosa was common in both cohorts (30% in both cohorts). (Figure 2) A clinical diagnosis of LCA (23% vs. 9%, *p* = .04) and Stargardt disease (9% vs. 0%, *p* = .02) was more common in the Indian cohort (Figure 2). Usher syndrome was diagnosed only in the US cohort, albeit in only four cases (0% vs. 7%, *p* = .03). The clinical diagnosis was uncertain, nonspecific, or being questioned in a substantial number of patients in both cohorts (India vs. US: 38% and 56%, *p* = .85).

Overall, testing led to a definitive molecular diagnosis in 68 cases (52%) and an indeterminate molecular diagnosis in 7 additional cases (5%). (Table 1, Figure 2) A definitive diagnosis was more likely in the India cohort as compared to the entire US cohort (62% vs. 39%, *p* = .009), whereas an indeterminate diagnosis was only seen in the US cohort (12%). (Table 1) Of note, one patient in the US cohort with a single LP variant in *GUCY2D* was categorized as negative. Although *GUCY2D* may cause autosomal recessive or dominant disease, the patient's phenotype was most consistent with autosomal recessive LCA (onset in infancy); therefore, this patient was considered to lack a definitive diagnosis.

The diagnosis rate among the six patients in the US cohort who were tested with <175 genes was slightly higher than those who were tested with the 175 gene panel (4/6; 66% vs. 25/51; 49%), respectively, indicating that testing for a small number of genes is not responsible for the lower diagnostic rate in the US cohort. Since some ethnic populations are underrepresented in genomic databases, we evaluated the differences in diagnosis rate among people of different ethnic backgrounds in the US cohort (Carss et al., 2017; Manrai et al., 2016). The non‐Hispanic Caucasian population (44 cases) had a definitive diagnosis in 19 cases (43%), an indeterminate diagnosis in 6 cases (14%), and no diagnosis in 19 cases (43%). The rate of definitive diagnosis in this group then is not statistically different from the Indian cohort (*p* = .06) and the rate of definitive and indeterminate diagnoses in Caucasians in the US cohort (57%) is similar to the Indian cohort. In contrast, the group of patients with Hispanic or non‐White ancestry in the US cohort (13 cases) had a definitive diagnosis in 3 cases (23%), an indeterminate diagnosis in 1 case (8%), and no diagnosis in 9 cases (69%). Although the numbers are small, the difference in definitive diagnosis is statistically different from the Indian cohort (*p* = .01) and the difference in definitive and indeterminate diagnosis approaches statistical significance (*p* = .06) supporting a lower diagnostic rate in underrepresented ethnicities (Carss et al., 2017).

Variants in 38 different genes were found in the 75 cases with a definitive or indeterminate molecular diagnosis, of these only 11 genes (*ABCA4*, *CEP290*, *CNGA3*, *CNGB3*, *CRB1*, *FZD4*, *GUCY2D*, *RDH12*, *RPE65*, *RPGRIP1*, and *USH2A*) had variants in both cohorts while the remaining 27 genes had variants in only 1 cohort. (Table S1) The most common causative gene was *GUCY2D* (6 patients), followed by *ABCA4* and *CRB1* (5 patients each). In the Indian cohort, 12 variants were novel, and in the US cohort, 26 variants were novel (including VUS). Among the 46 cases from India with a molecular diagnosis, 35 were consistent with autosomal recessive inheritance (76%), 7 were autosomal dominant (15%), and 4 were X‐linked (9%). Among the 29 cases with a definitive or indeterminate molecular diagnosis from the US, 22 were consistent with autosomal recessive inheritance (76%), 5 were autosomal dominant (17%), and 2 were X‐linked (7%). Consistent with a more frequent clinical diagnosis of possible LCA, a higher proportion of cases from India had a definitive molecular diagnosis of LCA compared to the US cohort (24% vs. 7%, *p* = .01). Retinitis pigmentosa was the most common diagnosis in both cohorts accounting for 30% of all clinical diagnosis (Table 1). A definitive or possible molecular diagnosis for retinitis pigmentosa was obtained in 64% (14/22) of cases in the Indian cohort while a definitive or possible molecular diagnosis was obtained in 53% (9/17) of cases in the US cohort. Mutations were observed in 13 different genes in the Indian cohort and 8 different genes in the US cohort with most of the genes being mutated in only one individual within each cohort. Only *USH2A* and *CERKL* genes were mutated in both cohorts. Overall, a definitive or possible molecular diagnosis was obtained in 80% (14/17) of LCA cases in the Indian cohort with mutations most commonly observed in *GUCY2D* (3 cases; 17%) while only 1 case (5%) of the mutations were noted in the *CEP290* gene (Table S1). Among the US cohort, a definitive or probable molecular diagnosis was obtained in 80% (4/5) of LCA cases with the most common mutations observed in the *CEP290* (3 cases; 60%) and *GUCY2D* genes (1 cases; 20%) (Table S1). We noted a significantly higher rate of homozygous variants in the India cohort as compared to the US cohort (61% vs. 12%; *p* < .0001). Of note, case 28 in the Indian cohort had a mutation in exon 15 of RPGR which we acknowledge is highly repetitive and, particularly in the middle, difficult to sequence by short read methods. However, at the edges of this region we feel that reads can be mapped sufficiently to confidently call variants.

Ten patients in the US cohort carried a single P or LP variant in an autosomal recessive gene consistent with carrier status. (Table S1) In five patients, there was no molecular diagnosis; the possibility that some or all of these five patients had a second undetected variant in the same gene cannot be excluded, such as deep intronic or promoter variants. The remaining five patients had a definitive or indeterminate molecular diagnosis within another gene. Carrier status was not reported in the Indian cohort. An additional 16 patients in the US cohort had one or more suspicious VUS; in two cases, the VUS was homozygous; in two other cases, there were two VUS in the same gene; and the remaining cases had one VUS in a gene. In the Indian cohort, 30 cases had one or more suspicious VUS; in 8 cases, the VUS was homozygous and in 8 cases there were two VUS in the same gene.

## 4 DISCUSSION

Despite the limitations of clinical testing, across the two cohorts, we had a definitive molecular diagnosis in 52% of cases, which is similar to the 50%–65% diagnostic rate in the literature (Audo et al., 2012; Glockle et al., 2014; O'Sullivan et al., 2012). However, the entire US cohort has a lower definitive diagnostic rate (39%) than the literature while the Indian cohort with a diagnostic rate of 62% had a higher diagnostic rate than many reports. Possible explanations for the differences in diagnostic rates across the two cohorts include (a) the inclusion of patients with diverse ethnic backgrounds as there was a lower diagnostic rate among non‐Caucasians in the US cohort (b) high consanguinity in the Indian cohort, and/or (c) different application of the ACMG criteria across the two institutions (Amendola et al., 2016).

Our study shows a lower diagnostic rate in Hispanic and/or non‐Caucasian populations. These racial/ethnic groups may be underrepresented in many population databases, locus specific disease databases, and disease‐gene association databases that may lead to both overdiagnosis and underdiagnosis of inherited disorders (Carss et al., 2017; Manrai et al., 2016). Manrai et al. (2016) have shown overdiagnosis of hypertrophic cardiomyopathy in black Americans due to underrepresention of this population's benign variants in databases. However, the Carss study showed underdiagnosis as evidenced by a decreased diagnostic rate in individuals of African ancestry but not those of South Asian ancestry (Carss et al., 2017).

The Indian cohort also detected a higher proportion of homozygous variants consistent with higher prevalence of consanguinity in South India (up to 24% in South India as compared to 16% in North India and 2% in Europeans) (Juyal et al., 2014; Srilekha et al., 2015). Retinitis pigmentosa, the most common form of blindness in the industrialized world, has a prevalence of between 1 in approximately 5,000 people in western populations (Bundey & Crews, 1984; Bunker, Berson, Bromley, Hayes, & Roderick, 1984). In contrast, a population‐based study by Sen et al showed the prevalence of retinitis pigmentosa in South India to be 1 in 930 people in urban areas and as high as 1 in 372 people in rural areas (Sen et al., 2008). Another population‐based study from rural Central India also showed a prevalence of retinitis pigmentosa (1:750 people) (Nangia, Jonas, Khare, & Sinha, 2012). This higher prevalence of retinitis pigmentosa in the Indian population was also confirmed in two hospital‐based studies (Kumaramanickavel, Joseph, Vidhya, Arokiasamy, & Shridhara, 2002; Ravi Babu & Nehakamalini, 2017). Thus, targeted screening for specific ophthalmic conditions and community‐based counseling efforts may play an important role in reducing the incidence of genetically transmitted ophthalmic disorders in India. In addition to specific ophthalmic conditions, the high consanguinity rate in South Indian population may have implications for community education and population screening for Mendelian disorders in general. Our results are also consistent with a previous report that showed that the prevalence of mutations in the *CEP290* gene (including the most common intronic variant) is significantly lower in the Indian population as compared to Western populations where the mutational frequency in *CEP290* is estimated to be account for ~ 20% of LCA cases (Daiger et al., 1998; Sundaresan et al., 2009). Despite the limited number of cases with LCA in the Indian cohort, we observed the frequency of mutations in *GUCY2D*, *AIPL1*, and *RPGR1P1* in the Indian cohort to be similar to what is described in published databases (Daiger et al., 1998). Although mutations in *SPATA7* were observed in 2 cases (11%) of the Indian cohort and no cases with *RPE65* mutation were observed in the Indian cohort, the limited sample size precludes additional assessment of mutational frequency in these genes in the Indian cohort. Similarly, although there may be some differences in diagnostic yield and mutation distribution in cases with clinical diagnosis of retinitis pigmentosa, the small sample sizes in this study precludes further assessment of mutation distribution between the Indian and US cohorts. Finally, differences in application of the ACMG criteria across the two institutions, which was shown to have only 71% reproducibility across laboratories even after training may account, in part, for the differences in definitive molecular diagnostic rates across the two institutions (Amendola et al., 2016). Our study has the limitations inherent in a retrospective clinical study: family may not be available for segregation studies or phase determination and phenotypes may not be well defined.

Clinical laboratories may obtain a similar diagnostic rate to the literature, although heterogeneity in racial background or consanguinity rates in the population being tested may lead to different diagnostic rates within individual populations. This information may be useful for counseling patients post genetic testing.

## CONFLICT OF INTEREST

The authors declare no conflict of interest.

## AUTHOR CONTRIBUTIONS

Study design: BT, RV. Data curation: JH, SY, SiM, AG, and AG. Methodology: RV, SaM, RG, JH, AG, and AG. Data analysis: SY, BT, SiM, and LS. Manuscript preparation: SY. Manuscript editing: SY, BT, LS, JH, AG, and RV.
